# Cohort Profile Update: The Young-HUNT Study, Norway

**DOI:** 10.1093/ije/dyae013

**Published:** 2024-02-01

**Authors:** Vegar Rangul, Turid Lingaas Holmen, Arnulf Langhammer, Jo Magne Ingul, Kristine Pape, Jørn Søberg Fenstad, Kirsti Kvaløy

**Affiliations:** HUNT Research Centre, Department of Public Health and Nursing, Faculty of Medicine and Health Sciences, Norwegian University of Science and Technology, Levanger, Norway; Levanger Hospital, Nord-Trøndelag Hospital Trust, Levanger, Norway; HUNT Research Centre, Department of Public Health and Nursing, Faculty of Medicine and Health Sciences, Norwegian University of Science and Technology, Levanger, Norway; Levanger Hospital, Nord-Trøndelag Hospital Trust, Levanger, Norway; HUNT Research Centre, Department of Public Health and Nursing, Faculty of Medicine and Health Sciences, Norwegian University of Science and Technology, Levanger, Norway; Levanger Hospital, Nord-Trøndelag Hospital Trust, Levanger, Norway; Regional Centre for Child and Youth Mental Health and Child Welfare, Department of Mental Health, Faculty of Medicine and Health Sciences, Norwegian University of Science and Technology, Trondheim, Norway; Department of Public Health and Nursing, Faculty and Medicine and Health Sciences, Norwegian University of Science and Technology, NTNU, Trondheim, Norway; HUNT Research Centre, Department of Public Health and Nursing, Faculty of Medicine and Health Sciences, Norwegian University of Science and Technology, Levanger, Norway; Levanger Hospital, Nord-Trøndelag Hospital Trust, Levanger, Norway; HUNT Research Centre, Department of Public Health and Nursing, Faculty of Medicine and Health Sciences, Norwegian University of Science and Technology, Levanger, Norway; Levanger Hospital, Nord-Trøndelag Hospital Trust, Levanger, Norway

**Keywords:** Trøndelag Health Study, Young-HUNT, adolescents, cohort profile update

Key Features

- Young-HUNT is the adolescent part of the Trøndelag Health Study (HUNT).
- In the Young-HUNT Study, all inhabitants aged 13–19 years, living in the northern part of Trøndelag, Norway, have been invited to repeated surveys since 1995–97. The study data may be linked to local and national health registries.
- The Young-HUNT4 Survey in 2017–19 included 8066 participants (76% of invited).
- The Young-HUNT4 data enable longitudinal follow-ups through repeated participations in the HUNT Study, and within-family studies.
- New measures include body composition analysis using bioelectrical impedance; a 1-week accelerometer recording on movement behaviour; and saliva for DNA samples.
- Researchers can apply for HUNT data access from HUNT Research Centre [https://www.ntnu.edu/hunt] after project approval from the Regional Committee for Medical and Health Research Ethics.

## The original cohort

The Young-HUNT Study is the adolescent part of the Trøndelag Health Study (The HUNT Study), a population-based health study inviting all inhabitants, aged 13 years and above, of primarily the northern part of Trøndelag County (former Nord-Trøndelag) in the central part of Norway. The HUNT study has collected data in four subsequent surveys every 11th year over 1984–2017,^1^ but collections of adolescent data, aged 13–19 years, started for the first time in 1995.

The first three waves of The Young-HUNT Study were described in the original cohort profile.^2^ The first survey, Young-HUNT1 (YH1), was performed in 1995–97 (*n* = 8983, response rate 88%) as a part of the HUNT2 Survey. The Young-HUNT2 9YH2) survey was a 4-year follow-up of the youngest Young-HUNT1 participants (13–15 years). The Young-HUNT3 (YH3) survey was performed in 2006–08 as part of the HUNT3 Survey (*n* = 8200, 78.4%).

**Figure 1.**
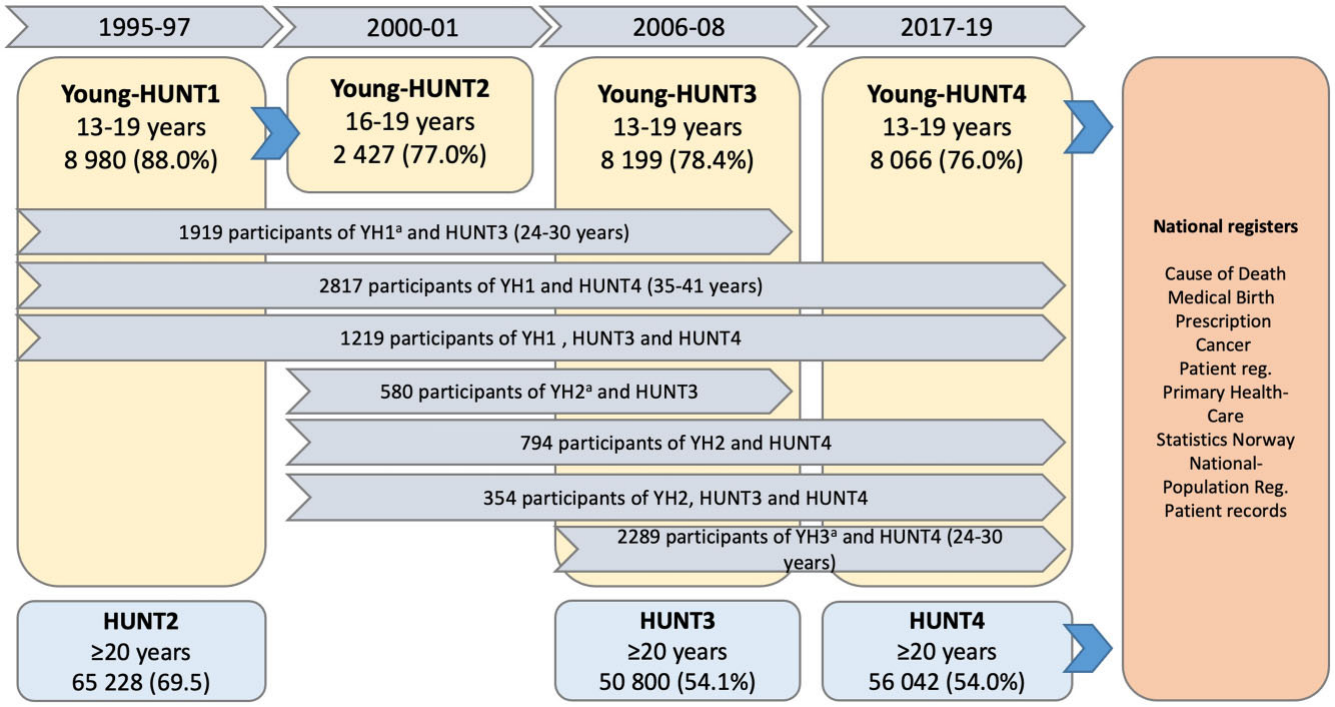
Flowchart of the Young-HUNT cohorts and participation in the adult survey waves (HUNT2, HUNT3 and HUNT4): cohort’s age range, sample size and response rate. ^a^YH1, Young-HUNT1 cohort; YH2, Young-HUNT2 cohort; YH3, Young-HUNT3 cohort

The Young-HUNT Study is a school-based survey primarily in the age range 13–19 years, and participant invitations are based on schools’ class lists of all registered school pupils, therefore also including a few 12- and 20-year-olds. As illustrated in Figure 1, The Young-HUNT allows longitudinal follow-up into adulthood, as well as linkage to a wide range of regional and national health registries by means of the unique identification number allocated to all Norwegian residents.^1^ All current residents ≥13 years of age in former Nord-Trøndelag have been invited to the surveys: HUNT2 (1995–97, 74 208 participants, 78.0%) and HUNT3 (2006–08, 58 999 participants, 70.1%).^3^

## What is the reason for the new data collection?

Since 1995, subsequent surveys have been carried out at 11-yearly intervals to enable tracking and revealing potential trends between adolescent cohorts. During the past decade there have been major societal changes, and it is important to uncover what influences the increasing incidences of mental health in youth populations.^4^^,^^5^ With increased life expectancy, and thereby rising prevalence of long-term adverse health conditions and multimorbidity, there is an urgent need for robust, longitudinal data to help understand the risk trajectory of age-related diseases and explore the effects of behavioural and biological determinants on a healthy life. The longitudinal data of approximately 2800 Young-HUNT participants into adulthood are of immense value for this purpose.

The need for a new data collection was related to several issues; First, repeated data collections are important for investigating trends over time and to get updates on prevalence and incidence of disorders and diseases across adolescent cohorts. Second, a new data collection will expand the opportunities for longitudinal follow-ups through the lifespan and for exploring causal pathways from adolescence to adulthood. Third, new participants can be linked to their family members in the HUNT database and contribute to larger intergenerational studies of inheritance on selected traits and health-related relationships. Last, the new data collection provide improved methodology with standardizes scales and objective measures for daily physical activity and body composition.

## What will be the new areas of research?

One of the largest societal experiments in modern times, the widespread use of smartphones and social media platforms probably plays a key role regarding health and wellbeing of the present adolescent generation.^6^ Hence, the potential impacts of this quite recent technology will be important to address in the coming years. In addition, new health updates provide the opportunity for new insights into the current mental health status and allow studies of determinants such as deliberate self-harm and suicidal behaviours to be explored. The opportunities for studying the long-term impact of early life health and behaviours are strengthened by adding new data that may also be linked to national registries and follow-up data collections.

More valid studies of physical activity and anthropometry are possible with the new and improved measurements including accelerometer recordings and body composition analyses using bioelectrical impedance. With the addition of device-based measurement of physical activity, a main research focus is to investigate the levels of physical activity intensity and energy expenditure during activities of daily living, and to conduct detailed analyses of sedentary behaviour such as sitting time, which may be influenced by novel developments of media use and digitalization. The complex interplay between physical activity, sedentary behaviour and body composition, all key health parameters, may be modelled in a much more precise and comprehensive manner than before.^7^ Additionally, the combined analyses applied to follow-up data from adolescence into adulthood provide opportunities to explore life course trajectories predictive of optimal health pathways.

Family is a main health determinant for adolescents, not only through the family environment but also through common genetic influences. Genotypic information from both parents and offspring in several generations represented in the HUNT Study allows for examining the contribution of genetics and direct or indirect inherited factors.^8^ The use of genetic data, combined with environmental information that is both in-depth and extensive, opens unique opportunities in this cohort.

## Who is in the cohort?

All pupils in the northern part of Trøndelag County, aged 13–19 years, were invited (in total 10 608 adolescents) to participate in Young-HUNT4 from August 2017 to January 2019, when 8066 (76%) participated (Table 1). The principals of all the 67 schools in the county gave their consent to their school’s participation. The lists of pupils in each class were the basis for the invitations. A screening station was established in each of the 67 schools included. Participation across the different study parts is shown in Table 1; all participants filled out the questionnaire, 7950 took part in the clinical investigation and 7852 participants completed the interview. The participation attendance was similar in both lower (8th–10th grade) and upper secondary schools (11th–13th grade). Apprentices and adolescents not in school, according to the registers of the school authorities, were invited to the survey by post. The questionnaire was included in the letter of invitation, along with invitation to attend the clinical examination at the study site nearest the pupil’s home. The Young-HUNT Study has a high attendance rate among adolescents attending school, but very low among those not attending school. Therefore, adolescents with potentially more adverse somatic and mental health problems might be underrepresented. Anyhow, adolescent health trends and prevalences identified in the Young-HUNT Study are generally representative of what is found in the national adolescent survey Ungdata.^9^

**Table 1.** Overview of themes measured in the Young-HUNT4 survey

Study part and data collection method	Selection criteria	Participants (% of invited)	Theme/content
Questionnaire	All pupils of northern part of Trøndelag (Nord-Trøndelag) who, during the period of screening, were students registered from 8th grade to the last year of upper secondary school	8066 (76.0%)	Lifestyle: exercise and physical activity, smoking habits, alcohol/drug use, nutrition, leisure time activities, social media/screen time. Mental health: anxiety/depression, self-esteem, personality, wellbeing, resilience, fatigue, internalized and externalized difficulties, bullying, disordered eating. Somatic health: disabilities, subjective health problems, psychosomatic disorders, eating habits. Somatic symptoms and diseases: allergy, respiratory disease, sleep disturbance, gastrointestinal diseases and symptoms, neurological disease, endocrine diseases. Medication: allergy, respiratory, gastrointestinal and other medication. Various: body image, puberty, network and neighbourhood, school, serious life events
At the examination stations			
Clinical measurements	All participants	7950 (75.0%)	Height, weight, body composition (bioelectrical impedance analysis)
Physical behaviour	All participants	5664 (54.6%)	Accelerometer sensors at the thigh and lower back for 1 week
Interview	All participants	7852 (74.8%)	Family (cohabit), urban/rurality, transport to school, parental occupational classification, headache/migraine
DNA (from saliva/Oragene)	All participants	7106 (68.5%)	Stored at room temperature until DNA extraction
Blood pressure	30% randomly selected participants	2550 (24.0%)	Systolic and diastolic pressure, pulse

Characteristics of Young-HUNT4 participants are given in Table 2. Participation in Young-HUNT4 does not differ between age and gender. Girls seems to have a wider waist circumference and percent body fat mass compared with boys, and boys have a higher physical activity level, both self-reported and objectively measured, than girls. There are clear gender differences in self-reported health, where girls have lower self-reported mental and somatic health, including a higher proportion of girl reporting loneliness compared with boys. The proportion of adolescents with risk behaviour such as daily smoking is incredibly low, and the proportion who drink alcohol and have been drunk several times is higher among boys compared with girls, with an increase with increasing age. Screen time use, 4 h or more per day, differs between girls and boys, where playing video games is more common among boys and girls are using social media.

**Table 2.** Characteristics of participants in the Young-HUNT4 Survey; stratified by sex and age group, percentages unless otherwise noted

Characteristics	All		Aged 13–15 years		Aged 16–19 years	
	Girls (*n*=3913)	Boys (*n*=3626)	Girls (*n*=1349)	Boys (*n*=1316)	Girls (*n*=2564)	Boys (*n*=2310)
Age, years, mean (SD, range)	16.1 (1.8, 12.7–21.8)	16.1 (1.8, 12.7–20.0)	14.4 (0.8, 12.7–15.9)	14.4 (0.9, 12.7–15.9)	17.5 (0.9, 16.0–21.8)	17.5 (1.0, 16.0–20.0)
BMI, kg/m^2^, mean (SD, range)	22.5 (4.2)	22.0 (4.3)	21.4 (3.7, 12.0–41.9)	20.7 (3.7, 13.6–41.8)	23.5 (4.3, 15.2–48.3)	23.2 (4.4, 15.1–56.4)
BMI category (IOTF)						
Underweight (<18.5 kg/m^2^)	5.4	5.8	6.8	6.8	4.7	5.2
Normal weight (18.5–24.9 kg/m^2^)	69.7	70.8	71.6	73.6	68.8	69.2
Overweight (25.0–29.9 kg/m^2^)	18.4	16.3	17.1	14.9	19.0	17.1
Obese (30.0–34.9 kg/m^2^)	4.9	5.3	3.9	4.0	5.4	6.0
Severely obese (≥35.0 kg/m^2^)	1.6	1.8	0.6	0.7	2.2	2.4
Waist circumference, cm (SD, range)	81.6 (11.8, 59.0–144.2)	79.5 (13.5, 58.8–151.0)	78.3 (10.5, 59.0–139.0)	74.8 (11.1, 58.8–143.5)	85.2 (11.9, 64.6–144.2)	84.2 (14.0, 63.2–151.0)
Percent body fat mass, % (SD)	27.7 (8.1)	16.7 (8.3)	25.7 (7.9)	16.7 (8.6)	29.5 (7.9)	16.7 (16.7)
Body fat mass, kg (SD)	17.8 (8.8)	12.0 (9.1)	15.5 (7.7)	10.6 (7.8)	19.9 (9.3)	13.4 (10.1)
Lean body mass^a^, kg (SD)	41.1 (5.4)	52.11 (10.2)	42.3 (5.5)	49.4 (9.6)	45.1 (5.6)	61.3 (8.6)
Systolic blood pressure, (mmHg)^b^^,^^c^	113.1 (9.2)	118.7 (11.1)	108.0 (9.0)	112.2 (9.9)	115.1 (8.7)	122.5 (10.0)
Smoking, %						
Never	92.5	89.0	97.6	96.8	88.1	82.4
Sometimes	3.6	6.2	1.1	0.8	5.8	10.9
Daily	0.3	0.5	0.1	0.3	0.5	0.7
Alcohol: occasionally/current						
No	52.2	55.4	84.6	86.9	24.4	28.6
Yes	47.8	44.6	15.4	13.1	75.6	71.4
Intoxicated more than 25 times	17.7	24.3	1.1	2.6	20.6	27.7
Physical activity frequency						
Less than once a week	16.1	15.2	11.5	10.7	20.0	19.0
1–3 days a week	51.5	43.8	49.8	44.7	53.0	43.0
4 days a week or more	32.4	41.0	38.7	44.5	27.0	38.0
Physical activity^d^, mean min/day (SD, range)	105 (33, 15.8–291)	114 (40, 10.7–292)	113 (34, 26.7–291)	122 (41, 15.1–292)	97 (29.8, 15.8–201)	105 (37, 10.7–235)
Somatic health: neither good or bad/bad	36.3	24.6	29.7	21.4	41.9	27.4
Mental health: neither good or bad/bad	40.3	20.5	32.6	17.7	46.8	23.0
Loneliness: often/very often	14.3	6.7	11.3	4.6	16.9	8.4
Fatigue (tiredness)						
More than 75% of the time	9.5	4.5	7.5	4.5	10.9	4.5
Often (except exercise)	64.2	40.8	47.6	34.2	72.9	44.6
More than 50% of the time	33.9	17.9	24.8	14.2	37.1	19.5
Self-harm: 2 times or more	18.3	5.3	6.8	2.3	11.4	3.1
Video games: 4 h or more per/weekday	15.4	24.0	16.9	24.8	14.3	23.3
Social media use: 4 h or more per/weekday	39.6	26.0	34.9	20.4	43.6	30.7

BMI, body mass index; IOTF, International Obesity Task Force (international BMI cut-offs).

a Lean body mass is the difference between total body weight and body fat weight.

b Mean of the 2nd and 3rd measurements.

c 30% of the participants (30% random sample of invited).

d Objectively measured with accelerometer. Active time is calculated as activities with a duration of over 60 s and is the sum of time walking, running and cycling. Participants with less than 10 min average active time per day were excluded. Participants with less than 3 days of active time were excluded.

## What has been measured?

Like previous Young-HUNT surveys, data were collected through a self-reported questionnaire, a short interview and clinical examinations (Table 3). Questionnaires were completed in the classroom setting, individually like a school examination, so that privacy was ensured. Young-HUNT4 questionnaire was a web form which gives the possibility to adapt questions to answers on previous questions, reduce non-response due to skip logic errors, and controls on questions to prevent obvious faulty registrations. Unlike the previous Young-HUNT surveys, sampling of biological material for DNA was through collection of saliva (Oragene, OG-500, DNA Genotek, Ottawa, Canada). The data collection procedure was thoroughly instructed by trained health professionals according to the manufacturer’s protocol. A pilot for the survey was carried out in a municipality outside the county of data collection. All parts of the survey were tested, and some of the instruments were adapted and adjusted according to experiences in this pilot, such as wording in some of the questions.

**Table 3.** Summary of the contents of the Young-HUNT1 (YH1), Young-HUNT2 (YH2), Young-HUNT3 (YH3) and Young-HUNT4 (YH4) surveys

Construct/theme	YH1	YH2	YH3	YH4
Population characteristics				
Age	x	x	x	x
Sex	x	x	x	x
Self-reported questionnaire				
Family	x	x	x	x
Family economy	x	x	x	x
Education	x	x	x	x
Household	x	x	x	x
Social contact, social support and loneliness				
Network and friends	x	x	x	x
Visiting someone/contact with friends	x	x	x	x
Participation in leisure time activities	x	x	x	x
Lifestyle factors				
Smoking	x	x	x	x
Alcohol usage	x	x	x	x
Narcotic/drugs usage		x		x
Exercise/physical activity	x	x	x	x
Sport participation	x	x	x	x
Nutrition and dieting	x	x	x	x
Screen-time/computer use			x	x
Use of social media/internet				x
Gaming				x
Gaming addiction				x
Sleep	x	x	x	x
Mental health				
Anxiety and depression symptoms	x	x	x	x
Self-esteem	x	x	x	x
Life satisfaction	x	x	x	
Resilience			x	x
Personality	x	x	x	x
Fatigue				x
Internalizing and externalizing problems				x
Bullying	x	x	x	x
Disordered eating	x	x	x	x
Physical and somatic health				
Atopic and respiratory symptoms and diseases	x	x	x	x
Disabilities	x	x	x	x
Psychosomatic symptoms and pain	x	x	x	x
Eating habits	x	x	x	x
Health at the moment	x	x	x	x
Health conditions	x	x	x	x
Dental health				x
Medications (prescribed and over the counter)	x	x	x	x
Pubertal development	x	x	x	x
Body image	x	x	x	x
Various health influencing factors				
Sexual orientation				x
Contraceptive usage	x	x	x	x
Sexual intercourse				x
School (teacher and classmate support, academic achievement)	x	x	x	x
Learning difficulties	x	x	x	x
Adverse life events			x	x
Health care use	x	x	x	x
Interview				
Headache/migraine	x	x	x	x
Social anxiety			x	
Family (cohabit)				x
Transport to school				x
Parental occupation				x
Measurements				
Height	x		x	x
Weight	x		x	x
Hip and waist circumference	x		x	x
Body composition/bioelectrical impedance analysis				x
Blood pressure	x		x	x
Heart rate	x		x	x
Spirometry (lung function)			x	x
Physical activity (objectively measured with accelerometer)				x
DNA				
Buccal swabs (stored on FTA cards)			x	
Saliva (Oragene kits)				x

FTA, Flinders Technology Associates.

The questionnaire included the core questions from earlier surveys, covering a broad range of topics concerning mental and somatic symptoms and diseases, health behaviour and lifestyle, and factors related to family, living conditions, school, leisure time and social life (Table 1).

Several novel items were added in Young-HUNT4 (YH4) which are highly relevant for adolescent health today and pinpointed as important exposure factors for future adult life. Mental health is measured by the Hopkins Symptom Checklist (HSCL) with five items included in YH1–YH3 (HSCL-5) increased to 11 items in YH4 (HSCL-5 and HSCL-10) .^10^ These instruments have been found to be valid and are used as in previous studies.^11^ Issues addressed in this respect are questions concerning challenging life events such as loneliness, trauma and violence, bullying and self-harm, complementary aspects related to mental health such as fatigue, internalizing and externalizing difficulties, gaming, networking, social media/screen time, sleep difficulties and gender- and age-specific topics such as puberty, pubertal development and sexual orientation (Supplementary Table S1, available as Supplementary data at *IJE* online).^12–16^ In addition, more standardized instruments were used to measure mental health [Strengths and Difficulties Questionnaire (SDQ) and HSCL] in Young-HUNT4 compared with previous Young-HUNT surveys.^17^

In addition to capturing leisure time and physical activities in questionnaires, physical activity, sedentary behaviours and sleep patterns were assessed using two accelerometers (AX3, Axivity, Newcastle, UK). The accelerometers were worn and attached to the thigh and lower back for 7 consecutive days, across the 24-h spectrum. The accelerometers were configured to record at 50 Hz and a range of ±8 g. Recording of at least 3 days was considered valid.

Improved aspects of the clinical examination in Young-HUNT4, compared with previous surveys, included body composition analysis through bioelectrical impedance analysis (BIA) (InBody 770, Cerritos, CA, USA) which measures how the body impedes electric flow through fat, muscle and blood, thereby giving a more accurate measure of body composition than previous manual measurements of, for instance, body mass index (BMI) and waist circumference for overweight and obesity categorization.

In 2550 (32%) randomly selected participants from both lower and upper secondary school, blood pressure, pulse and peripheral capillary oxygen saturation were recorded three times in 1-min intervals using Dinamap CARESCAPE V100 (GE Healthcare, Chicago, IL, USA).

## What has it found? Key findings and publications

To date approximately 150 publications have used data from the Young-HUNT Study. Important findings influenced planning of data collection in the Young-HUNT4 Survey. In Table 2 we present descriptive characteristics of the Young-HUNT4 participants, including prevalence estimates of a range of somatic and mental conditions, in addition to lifestyle behaviours. There has been an alarming increase in the prevalence of obesity and overweight over the past decennials in the Western world and in the Nordic countries.^18^^,^^19^ In agreement with these findings, we see a remarkable high average waist circumference (WC) among adolescents both aged 13–15 years and aged 16–19 years, with a higher mean WC among girls (Table 2) compared with reference values in Norwegian adolescents.^20^ In contrast, we see no sex difference in overweight and obesity defined by BMI in Young-HUNT4. Along with a general weight increase in the population,^21^ underestimation of overweight has been shown to increase among adolescents,^22^ which may prohibit weight-reducing efforts. Positive body perception, however, is associated with better mental health and prevents weight increase into adulthood.^23^

Most adolescents have levels of physical activity that fulfil the national recommendations, with a mean daily physical activity level above 100 min (active time is objectively measured by accelerometer and calculated as activities with a duration of over 60 s, and is the sum of time with walking, running and cycling). Among the Young-HUNT4 participants, boys are more physically active than girls are at all ages, and the youngest adolescents are more physically active than the older ones (Table 2). The physical activity level in adolescence, and body image and eating behaviours, seem not to be clearly associated,^24^ although the ones who considered themselves overweight where less likely to be physically active compared with those with normal weight. The association between physical activity and mental health was demonstrated in Young-HUNT3 (2006–08),^25^ and further confirmed in Young-HUNT4 (2017–19).^26^

Mental health problems have increased over the past decades in many adolescent populations.^27^ Considering all HUNT participants from age 13 years and upward, a divergent trend in mental distress was evident, with a sharp increase among adolescents and young adults in contrast to participants aged 60+.^28^ In Young-HUNT4, the prevalence of anxiety and depression symptoms above the recommended HSCL-5 scale cut-off was 16.5% for boys and 44.4% for girls. Increasing use of social media as a daily activity has led to an emergency impact on people’s lives. Screen time in total, and particularly prevalence in self-reported use of social media and video games, is high. Among Young-HUNT4 participants, girls spend their time on social media and boys spend their time gaming (Table 2). Long-lasting screen time (4 h or more per/day) among adolescents aged 13–19 is associated with mental distress^29^ and seems to be associated with poorer quality of life.^30^ This association was even stronger for more than 7 h/day. There was also an association between high screen time (≥7 h daily use) and loneliness.

The new focus on mental health, screen time and use of electronic devices has resulted in a study exploring sleep duration, prevalence of insomnia and use of electronic devices in bed (relation to sleep) among those with mental distress. Adolescents with mental distress (SCL-10, ≥1.85) seem to report short sleep duration and insomnia, and higher electronic device use in relation to sleep.^31^

## What are the main strengths and weaknesses?

The Young-HUNT Study is valuable for trend investigations, due to repeated surveys of the new cohorts of the same population since the 1990s. By linkage to follow-up data in HUNT and national registries, life course studies may be performed. Family linkage between Young-HUNT participants, within a total sample size of 240 000 HUNT participants, gives unique opportunities for intergenerational and sibling studies. Further, the survey has high participation rate, represents an ethnically quite homogeneous population, and data may be linked to valid information on migration, diseases and deaths, from registers. The HUNT Study covers a broad range of health-related topics, and many questionnaire items have been kept unchanged across the surveys to enable health and risk factor trends to be studied. Genetic material is available from Young-HUNT3 and Young-HUNT4 participants. Quality-controlled HUNT data are securely managed by the HUNT Databank and biological material (saliva) is stored in the state-of-the-art HUNT Biobank at the HUNT Research Centre.

A weakness of the survey is the low participation rate among adolescents not in school and apprentices (10% participation). This may affect the estimation of health problems and important behavioural patterns of these groups and bias the results in the total adolescent sample. The northern part of Trøndelag consists of mostly rural areas and has no large cities. It may well not be representative of populations living in large Norwegian cities. Confirming health trends identified in other Norwegian adolescent surveys, however, ensure that the Young-HUNT4 Survey is generalizable to other areas (rural and urban) in Norway and also probably to other adolescent cohorts in Europe.

## Can I get hold of the data? Where can I find out more?

Any research group with a principal investigator affiliated with a Norwegian research institution can apply for access to HUNT data. This means that researchers not affiliated to a Norwegian research institution may collaborate with and apply through a Norwegian researcher to use HUNT material. All projects must be approved by the Regional Committee for Medical and Health Research Ethics (REC) in Norway.

Certain data may be subject to a time-limited exclusivity provided to the researchers who have financed and conducted the data collection in specific scientific areas. Information concerning biological material and specific analytical services may be found at the HUNT Biobank website [https://www.ntnu.edu/hunt/hunt-biobank]. Linkages between HUNT data and registry data are obtained separately for each research project and require approval from REC and each registry owner. Detailed information on the application process and conditions for access is available at the HUNT website [https://www.ntnu.edu/web/hunt/data]. For queries, please e-mail Kirsti Kvaløy at [kontakt@hunt.ntnu.no].

## Ethics approval

Ethical approval for this study has been obtained from REC, license number: HUNT4 17/00426–7/GRA. HUNT4 was not processed and approved by REC as a normal project, as it was assessed as outside REC's mandate. HUNT is to be considered a health register and not a health research project, but REC has given feedback on the setup of the HUNT data collection and the design of the consent form.

## Supplementary Material

dyae013_Supplementary_Data

## Data Availability

See ‘Can I get hold of the data?’ above.

## References

[1] Asvold BO , Langhammer A, Rehn TA et al Cohort profile update: the HUNT study, Norway. Int J Epidemiol 2023;52:e80–91. 35578897 10.1093/ije/dyac095 PMC9908054

[2] Holmen TL , Bratberg G, Krokstad S et al Cohort profile of the Young-HUNT Study, Norway: a population-based study of adolescents. Int J Epidemiol 2014;43:536–44. 23382364 10.1093/ije/dys232

[3] Krokstad S , Langhammer A, Hveem K et al Cohort profile: the HUNT study, Norway. Int J Epidemiol 2013;42:968–77. 22879362 10.1093/ije/dys095

[4] Bor W , Dean AJ, Najman J, Hayatbakhsh R. Are child and adolescent mental health problems increasing in the 21st century? A systematic review. Aust N Z J Psychiatry 2014;48:606–16. 24829198 10.1177/0004867414533834

[5] Erskine HE , Baxter AJ, Patton G et al The global coverage of prevalence data for mental disorders in children and adolescents. Epidemiol Psychiatr Sci 2017;26:395–402. 26786507 10.1017/S2045796015001158 PMC6998634

[6] Bozzola E , Spina G, Agostiniani R et al The use of social media in children and adolescents: scoping review on the potential risks. Int J Environ Res Public Health 2022;19:19. 10.3390/ijerph19169960 PMC9407706 36011593

[7] Carson V , Tremblay MS, Chaput JP, Chastin SFM. Associations between sleep duration, sedentary time, physical activity, and health indicators among Canadian children and youth using compositional analyses. Appl Physiol Nutr Metab 2016;41:S294–302. 27306435 10.1139/apnm-2016-0026

[8] Kong A , Thorleifsson G, Frigge ML et al The nature of nurture: Effects of parental genotypes. Science 2018;359:424–28. 29371463 10.1126/science.aan6877

[9] Yu BK , Von Soest T, Nes RB. Do municipal contexts matter for adolescent mental health? A within-municipality analysis of nationwide Norwegian survey data across six years. *Res Child Adoles Psychopathol* 2023. 10.1007/s10802-023-01123-3 37688765

[10] Strand BH , Dalgard OS, Tambs K, Rognerud M. Measuring the mental health status of the Norwegian population: a comparison of the instruments SCL-25, SCL-10, SCL-5 and MHI-5 (SF-36). Nord J Psychiatry 2003;57:113–18. 12745773 10.1080/08039480310000932

[11] Haavet OR , Sirpal MK, Haugen W, Christensen KS. Diagnosis of depressed young people in primary health care—a validation of HSCL-10. Fam Pract 2011;28:233–37. 20937663 10.1093/fampra/cmq078

[12] Askeland KG , Boe T, Breivik K, La Greca AM, Sivertsen B, Hysing M. Life events and adolescent depressive symptoms: protective factors associated with resilience. PLoS One 2020;15:e0234109. 32502163 10.1371/journal.pone.0234109 PMC7274383

[13] Bryant A , Guy J, Team C, Holmes J; CALM Team The strengths and difficulties questionnaire predicts concurrent mental health difficulties in a transdiagnostic sample of struggling learners. Front Psychol 2020;11:587821. 33329246 10.3389/fpsyg.2020.587821 PMC7717974

[14] Jozefiak T , Larsson B, Wichstrom L, Mattejat F, Ravens-Sieberer U. Quality of Life as reported by school children and their parents: a cross-sectional survey. Health Qual Life Outcomes 2008;6:34. 18489777 10.1186/1477-7525-6-34 PMC2409303

[15] Loge JH , Ekeberg O, Kaasa S. Fatigue in the general Norwegian population: Normative data and associations. J Psychosom Res 1998;45:53–65. 9720855 10.1016/s0022-3999(97)00291-2

[16] Solberg ME , Olweus D. Prevalence estimation of school bullying with the Olweus Bully Victim Questionnaire. Aggressive Behav 2003;29:239–68.

[17] Strand BH , Dalgard OS, Tambs K, Rognerud M. Measuring the mental health status of the Norwegian population: a comparison of the instruments SCL-25, SCL-10, SCL-5 and MHI-5 (SF-36). Nord J Psychiatry 2003;57:113–18. 12745773 10.1080/08039480310000932

[18] Buoncristiano M , Spinelli A, Williams J et al Childhood overweight and obesity in Europe: Changes from 2007 to 2017. Obes Rev 2021;22(Suppl 6):e13226. 34378305 10.1111/obr.13226

[19] Hemmingsson E , Ekblom O, Kallings LV et al Prevalence and time trends of overweight, obesity and severe obesity in 447,925 Swedish adults, 1995-2017. Scand J Public Health 2021;49:377–83. 32349623 10.1177/1403494820914802 PMC8135248

[20] Brannsether B , Roelants M, Bjerknes R, Juliusson PB. Waist circumference and waist-to-height ratio in Norwegian children 4-18 years of age: reference values and cut-off levels. Acta Paediatr 2011;100:1576–82. 21627692 10.1111/j.1651-2227.2011.02370.x

[21] Midthjell K , Lee CM, Langhammer A et al Trends in overweight and obesity over 22 years in a large adult population: the HUNT Study, Norway. Clin Obes 2013;3:12–20. 23935708 10.1111/cob.12009 PMC3734732

[22] Kvaloy K , Sandsgard-Hilmarsen E, Eik-Nes TT, Bratberg GH. Underestimation of Overweight and Health Beneficial Outcomes in Two Adolescent Cohorts in Norway—The HUNT Study. J Adolesc Health 2021;69:82–89. 33288462 10.1016/j.jadohealth.2020.10.026

[23] Cuypers K , Kvaloy K, Bratberg G, Midthjell K, Holmen J, Holmen TL. Being normal weight but feeling overweight in adolescence may affect weight development into young adulthood-an 11-year followup: the HUNT study, Norway. J Obes 2012;2012:601872. 22666556 10.1155/2012/601872 PMC3362140

[24] Krogsrud JC. Distribution of objectively measured physical activity among adolescents related to body image and eating behaviours: data from Young-HUNT4. Master Thesis. Norwegian University of Science and Technology, 2023.

[25] Guddal MH , Stensland SO, Smastuen MC, Johnsen MB, Zwart JA, Storheim K. Physical activity and sport participation among adolescents: associations with mental health in different age groups. Results from the Young-HUNT study: a cross-sectional survey. BMJ Open 2019;9:e028555. 10.1136/bmjopen-2018-028555 PMC6731817 31488476

[26] Bjerkan M , Rangul V, Skjesol K, Ulstad SO. Physical activity and depression/anxiety symptoms in adolescents—the young-HUNT study. Physical Activity and Health 2022;6:73–85.

[27] Ceccarelli C , Prina E, Muneghina O et al Adverse childhood experiences and global mental health: avenues to reduce the burden of child and adolescent mental disorders. Epidemiol Psychiatr Sci 2022;31:e75. 36245402 10.1017/S2045796022000580 PMC9583628

[28] Krokstad S , Weiss DA, Krokstad MA et al Divergent decennial trends in mental health according to age reveal poorer mental health for young people: repeated cross-sectional population-based surveys from the HUNT Study, Norway. BMJ Open 2022;12:e057654. 10.1136/bmjopen-2021-057654 PMC9119156 35584877

[29] Korpås JA , Screen usage associated with mental health among adolescents: Knowledge from a population-based study. Master Thesis. Norwegian University of Science and Technology, 2020.

[30] Førlandsås K , Screen usage associated with quality of life, and loneliness among adolescents in Trøndelag—a cross-sectional study based on Young-HUNT4 (2017–2019). Master Thesis. Norwegian University of Science and Technology, 2022.

[31] Muri EJ , Mapping sleep among adolescents with and without mental health distress—a cross-sectional study based on Young-HUNT4. Master Thesis. Norwegian University of Science and Technology, 2023.

