# Supplementary material for: Cohort Profile Update: The Young-HUNT Study, Norway
Source: Int J Epidemiol. 2024 Feb 1;53(1):dyae013. doi: 10.1093/ije/dyae013 (PMC10834360; doi:10.1093/ije/dyae013)
Supplement: dyae013_Supplementary_Data [file dyae013_supplementary_data.docx]

**Supplementary Table S1.** New instruments included in Young-HUNT4 to measure challenging life events, mental health, loneliness, trauma, bullying, screen-time (social media/internet, gaming) and network/neighborhood.

| **Construct/theme** | **Variable/Question** | **Description** | **Ref.** |
| --- | --- | --- | --- |
| Loneliness | At school or during your spare time. How often do you feel?   - That you miss socializing with others - That you are lonely | Two questions measuring loneliness.  5 categories, 1= Very seldom or never, 2 = Seldom, 3 = Sometimes, 4 = Often, 5 = Very often. Derived from Hughes et al., 2004. | ^1^ |
| Screen-time/watching TV, screen-based entertainment | In your spare time, how many hours per day do you spend watching TV or other screen-based entertainment? | The question originates from the WHO^a^ collaborative cross-national HBSC^b^ study in Europe. Wording in the question text or question choice may have been changed to better fit The Young HUNT Study. | ^2^ |
| Screen-time/ social media/chatting | In your spare time, how many hours per day do you spend on social medias or surfing/chatting online |  |  |
| Screen-time/ Gaming | In your spare time, how many hours per day do you play video games (on PC^c^, game console, tablet, cell phone, etc.) |  |  |
| Gaming addiction | Regarding video games and gaming, how much do you agree with the following statements?   - I use way too much time on video games - I get in a bad mood when I am unable to spend time on gaming - My parents tell me that I spend too much time gaming.   Regarding internet surfing and chatting, how much do you agree with the following statements?   - I use way too much time on internet communication and surfing - I get in bad mood when I am unable to spend time on internet communication and surfing - My parents tell me that I spend too much time on internet communication and surfing | The Aarhus School Survey questionnaire (Aarhus; Rasmussen, et al., 2015) is a six-item questionnaire intended to measure the respondents' own perceptions of computer gaming and Internet use. The questionnaire was developed by Rasmussen and colleagues for the Aarhus School Survey, Denmark in 2009. The participants rate how much they agree or disagree with six statements regarding computer gaming and internet usage on a six-point Likert Rating scale from '1 = Strongly agree' to '5 = Strongly disagree'. The psychometric properties of the Aarhus questionnaires are limited (as of May 2022), but a study by Holstein and colleagues (2014) found Cronbach's alpha > 0.7 for both dimensions. | ^3, 4^ |
| Inventory of life and quality | Think about how the last week has been:   - How well do you deal with the demands from school? - How good is your relationship with other family members (parents, siblings)? - How do you get along with peers in your spare time? - To what extent can you independently activate yourself (interests and hobbies, activities)? - How would you rate your bodily health condition? - How would you rate your mental health condition? - If you summarize all of the mentioned relationships and areas of your life. How are you, all in all? | The quality of life was measured using ILC, developed by Mattejat & Remschmidt (1998;2006). Das Inventar zur Erfassung der Lebensqualität bei Kindern und Jugendlichen (ILK). Bern: Hans Huber Verlag). The translation and back translation into German has been approved by the original authors and was conducted by Thomas Jozefiak (Jozefiak, Larsson, Wichstrøm, Mattejat and Ravens-Sieberer (2008) Quality of Life as reported by school children and their parents: a cross sectional survey. Health and Quality of Life Outcomes,6:34). The Norwegian version of the ILK consists of 3 questions and is thereby a short, reliable and valid instrument. | ^5, 6^ |
| Resilience | How have you thought or felt about yourself, and about your family during the last month?   - In my family we share views of what is important in life - I easily find new friends - I feel comfortable with my family - I am good at talking to new people - My family views the future as positive, even when very sad things happen - I always find something fun to talk about - I know how to reach my goals - I feel I'm skilled - In adversity, I tend to find something good to help me grow | An eight-item scale was selected based on extensive statistical procedures from the Resilience Scale for Adolescents (READ) where the original version contains 28 items. The READ is based on the previously developed Resilience Scale for Adults (RSA;33 items). The selected READ items belong to the following two factors:  Social competence (4 items) Family cohesion (4 items) Factors not included in HUNT were Personal competence, Structured style and Social resources. | ^7, 8^ |
| Fatigue | Do you often feel tired/exhausted (apart from after exercising)?  Approximately how long have you felt tired/exhausted?  Approximately how much of the time do you feel tired/exhausted? | Self-reported scale developed to measure the severity of fatigue. The questionnaire has 11 items and was developed to assess severity of fatigue symptoms. The seven first items measure physical fatigue, and the four last items measure mental fatigue. Three of these items were selected in The Young-HUNT4 Study. | ^9^ |
| Bullying | How many times has this happened to you in the last 6 months?   - I've been bullied, hit, got my hair pulled, kicked or attacked by peers - I've been isolated by peers, and I'm not allowed to join them - I've received unpleasant messages or pictures on my cell phone or via internet - I've bullied others - I’ve been made fun of, teased in a hurtful way by peers, or someone has said ugly things to me | Questions derived from different questionnaires concerning bullying among students (The Olweus Bully/Victim Questionnaire, and Slonje, R. & Smith, P.K. (2008) Cyberbullying: Another main type of bullying? | ^10^ |
| Interpersonal violence, brief lifetime trauma screen | Have any of the following things happened to you?   - That you or someone in your family has been seriously ill - Death of a loved one - A catastrophe (fire, hurricane, etc.) - A serious accident (e.g. a very serious car accident) - Subjected to violence (beaten/injured) by others - Seen others violently hurt - Been subjected to sexually uncomfortable/abusive acts by someone about your age - Been subjected to sexually uncomfortable/abusive acts by an adult - Experienced something else that was very frightening, dangerous or violent - Subjected to violence (beaten/injured) by someone close to me | The questions in Young-HUNT brief lifetime trauma screen regarding interpersonal violence was derived from the University of California at Los Angeles Post-traumatic Stress Disorder Reaction Index (UCLA PTSD Reaction Index) and adapted to the Norwegian context (e.g. neighborhood shootings are not very frequent in Norway, and were therefore left out of the questionnaire). The original formulation of the questions regarding sexual abuse was not approved by Regional Committees for Medical and Health Research Ethics (REC) since they were too direct and could lead to criminalization of individuals. They were therefore rewritten to more unspecific questions to suit the demands of HUNT and REC.The UCLA PTSD Reaction Index was developed from the Survey of Children’s Exposure to Community Violence (Kadra et al., 2014). | ^11, 12^ |
| Mental Health (symptoms of anxiety and depression) | In the last 14 days, have you been bothered by any of these?   - Felt afraid and anxious - Felt tense or uneasy - Felt hopelessness when you think of the future - Felt dejected or sad - Worried too much about various things - Suddenly been scared for no reason - Felt faintness, dizziness, or weakness - Blaming yourself for things - Had sleeping problems - Had a feeling of being useless, of little worth - Feelt that everything is an effort | SCL-10 is a short version of the Symptoms Checklist-25.  SCL-10 consists of ten items from SCL-25 and is designed to identify symptoms of anxiety and depression. The ten items used were selected on basis of a regression analysis of a study using items from SCL-25. Reliability of the SCL-instruments in a Norwegian population are tested in a random sample of 10 000 participants from Statistics Norway’s (SSB) Survey of Level of Living 1998. | ^13-15^ |
| Network and neighborhood | Do you have a steady boyfriend/girlfriend?  About how many close friends do you have?  How often have you done any of these activities?   - Hang out with friends after school hours. - Have contact with friends via internet.   Who do you usually live with:   - Both parents - Both parents, but shared - One of the parents - Other caregivers - Live in institution - Live in dorm - Live alone without caregivers - Live with mother - Live with father - Live with grandparents - Live with other relatives - Live with other adults   How far is the distance from where you live most of your time to:   - Closest neighbour - Closest friend - Closest store - School | These questions were suggested by Tambs and Moum, Norwegian Institute of Public Health, for The Young-HUNT Study. The questions are inspired by similar questions from other health studies but are selected and discretionary formulated without support in available psychometric data.  Some of these variables were collected through an interview. |  |

^a^ WHO, World Health Organisation ^b^ HBSC, Health Behaviour in School-aged Children ^c^ PC, Personal computer

**References**

1. Hughes ME, Waite LJ, Hawkley LC, Cacioppo JT. A Short Scale for Measuring Loneliness in Large Surveys: Results From Two Population-Based Studies. *Res Aging* 2004; **26**: 655-72.

2. HBSC. [cited; Available from: <www.hbsc.org>

3. Holstein BE, Pedersen TP, Bendtsen P, et al. Perceived problems with computer gaming and internet use among adolescents: measurement tool for non-clinical survey studies. *Bmc Public Health* 2014; **14**.

4. Rasmussen M, Meilstrup CR, Bendtsen P, et al. Perceived problems with computer gaming and Internet use are associated with poorer social relations in adolescence. *Int J Public Health* 2015; **60**: 179-88.

5. Mattejat F, Remschmidt H. [The assessment of therapy outcome in child and adolescent psychiatry under naturalistic conditions--conception and implementation of the Marburg System of Quality Assurance and Therapy Evaluation]. *Z Kinder Jugendpsychiatr Psychother* 2006; **34**: 445-54.

6. Jozefiak T, Larsson B, Wichstrom L, Mattejat F, Ravens-Sieberer U. Quality of Life as reported by school children and their parents: a cross-sectional survey. *Health Qual Life Outcomes* 2008; **6**: 34.

7. Hjemdal O, Friborg O, Stiles TC. Resilience is a good predictor of hopelessness even after accounting for stressful life events, mood and personality (NEO-PI-R). *Scand J Psychol* 2012; **53**: 174-80.

8. Hjemdal O, Vogel PA, Solem S, Hagen K, Stiles TC. The relationship between resilience and levels of anxiety, depression, and obsessive-compulsive symptoms in adolescents. *Clin Psychol Psychother* 2011; **18**: 314-21.

9. Chalder T, Berelowitz G, Pawlikowska T, et al. Development of a fatigue scale. *J Psychosom Res* 1993; **37**: 147-53.

10. Olweus D, Limber SP. Bullying in School: Evaluation and Dissemination of the Olweus Bullying Prevention Program. *Am J Orthopsychiat* 2010; **80**: 124-34.

11. Steinberg AM, Brymer MJ, Decker KB, Pynoos RS. The University of California at Los Angeles Post-traumatic Stress Disorder Reaction Index. *Curr Psychiatry Rep* 2004; **6**: 96-100.

12. Kadra G, Dean K, Hotopf M, Hatch SL. Investigating exposure to violence and mental health in a diverse urban community sample: data from the South East London Community Health (SELCoH) survey. *PLoS One* 2014; **9**: e93660.

13. Derogatis LR, Lipman RS, Rickels K, Uhlenhuth EH, Covi L. The Hopkins Symptom Checklist (HSCL): a self-report symptom inventory. *Behav Sci* 1974; **19**: 1-15.

14. Tambs K, Moum T. How Well Can a Few Questionnaire Items Indicate Anxiety and Depression. *Acta Psychiat Scand* 1993; **87**: 364-7.

15. Strand BH, Dalgard OS, Tambs K, Rognerud M. Measuring the mental health status of the Norwegian population: a comparison of the instruments SCL-25, SCL-10, SCL-5 and MHI-5 (SF-36). *Nord J Psychiatry* 2003; **57**: 113-8.
